# Development of a Questionnaire for Dental Utilization Based on the Andersen Model

**DOI:** 10.7759/cureus.66671

**Published:** 2024-08-12

**Authors:** Prachi, Sibyl Siluvai, Indumathi KP, Krishnaprakash G, Raghulgandhi Venkatesan, Darshana Bennadi

**Affiliations:** 1 Public Health Dentistry, SRM Kattankulathur Dental College and Hospital, SRM Institute of Science and Technology, Chengalpattu, IND; 2 Centre for Statistics, SRM Institute of Science and Technology, Chengalpattu, IND; 3 Public Health Dentistry, Sri Siddhartha Dental College and Hospital, Sri Siddhartha Academy of Higher Education, Tumkur, IND

**Keywords:** reliability, validity, dental utilization, andersen model, questionnaire development

## Abstract

Background and aim

Oral health is an integral part of overall general health. Understanding the variables that affect the use of dental services can help overcome the challenges and lessen disparities in oral health. There are no significant reliable questionnaires available for determining factors that influence dental care utilization. This study aimed to develop and validate a questionnaire based on the Andersen model of healthcare utilization to assess these factors.

Materials and methods

A preliminary questionnaire consisting of 34 items was developed based on the Andersen model of healthcare utilization. The questionnaire’s final version consisted of 24 items after five subject area experts analyzed its face validity and content validity. Content validity ratio (CVR) was used to evaluate the validity of the questionnaire. The Cronbach’s alpha coefficient was then used to establish the internal consistency and test-retest for the reliability of the questionnaire.

Results

The final version of the questionnaire included 24 items based on CVR, and the data of the final version suited the model well. The internal consistency measured with Cronbach’s alpha coefficient was above the threshold for each item. The Cronbach’s alpha value for test-retest reliability was found to be 0.72, which was acceptable.

Conclusions

The current results show that the proposed questionnaire for dental utilization based on the Andersen model has satisfactory validity and reliability. This questionnaire can be used as an instrument to determine the various factors that affect the utilization of dental services.

## Introduction

A vital component of overall general health is oral health, which affects many facets of daily living, including speaking, chewing, looking good, and connecting with society. Among the major public health issues, oral diseases are the fourth most expensive disease in developed nations [1]. The percentage of the population that uses dental health services over a given time is known as dental care utilization. There are several reports that most dental patients only choose to come back for follow-up care when they are in excruciating pain. To improve oral health outcomes, a thorough understanding of the factors influencing people’s behavior and how they use health services is required [2]. The Andersen model was formulated in the 1960s, which is a framework designed to determine the factors that either facilitate or impede healthcare utilization. It is a behavioral model that offers metrics for healthcare access. Three factors are thought to influence a person’s ability to access and utilize health services: the predisposing factor, the enabling factor, and the need factor [3].

A questionnaire is a frequently used health evaluation tool that assists in determining if a patient is making progress or not in achieving particular goals. They are frequently used to measure patient’s impressions of numerous elements of healthcare and capture the self-reported observations of the individual. How to create and build these questionnaires for use in the medical area is still not entirely clear [4]. This article aimed to connect these factors and develop a questionnaire for dental utilization based on the Andersen model, thereby identifying the most common barriers to dental care utilization.

## Materials and methods

**Figure 1:**
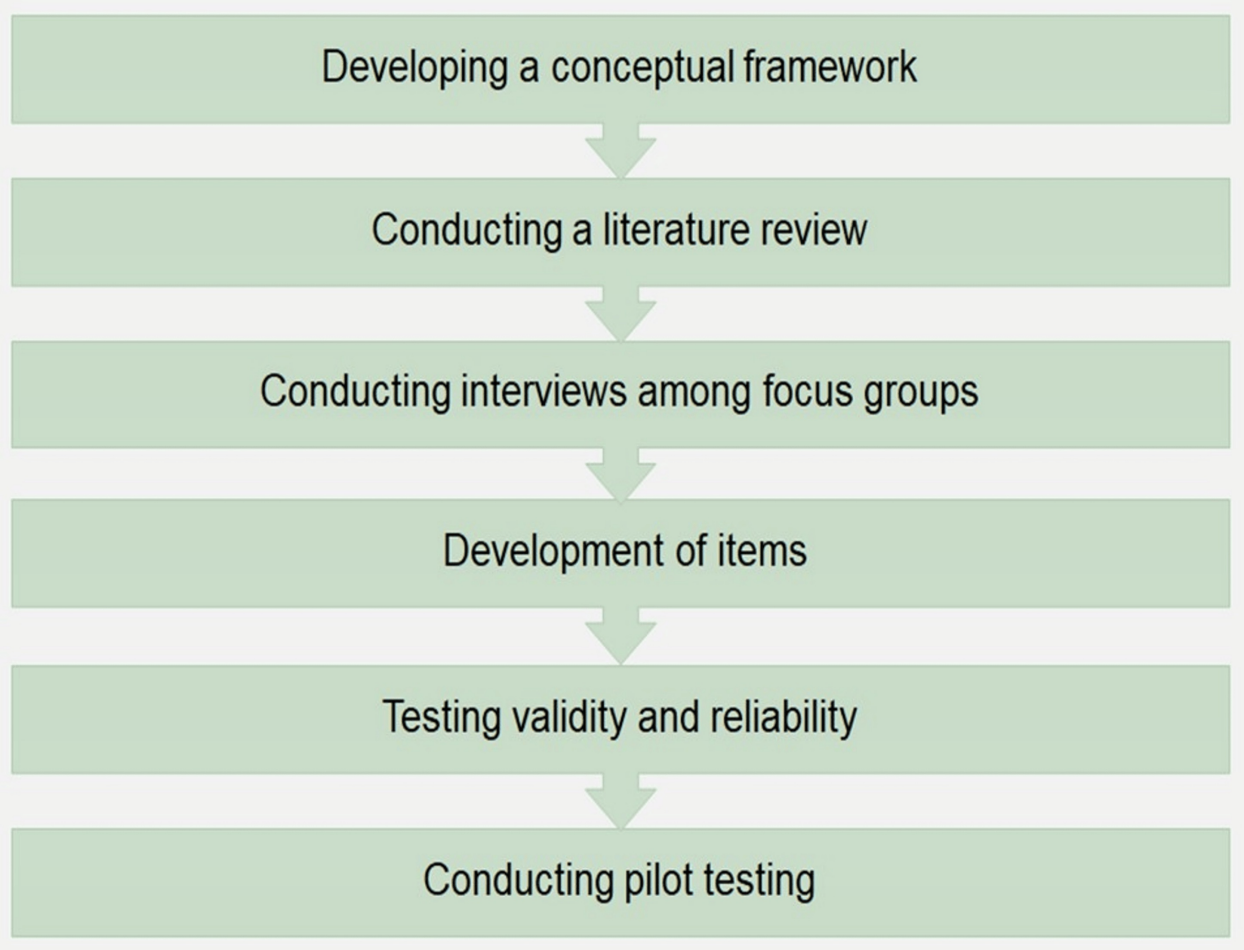
Steps in developing the questionnaire

The questionnaire was developed based on the following steps (Figure 1).

Developing a conceptual framework

**Figure 2:**
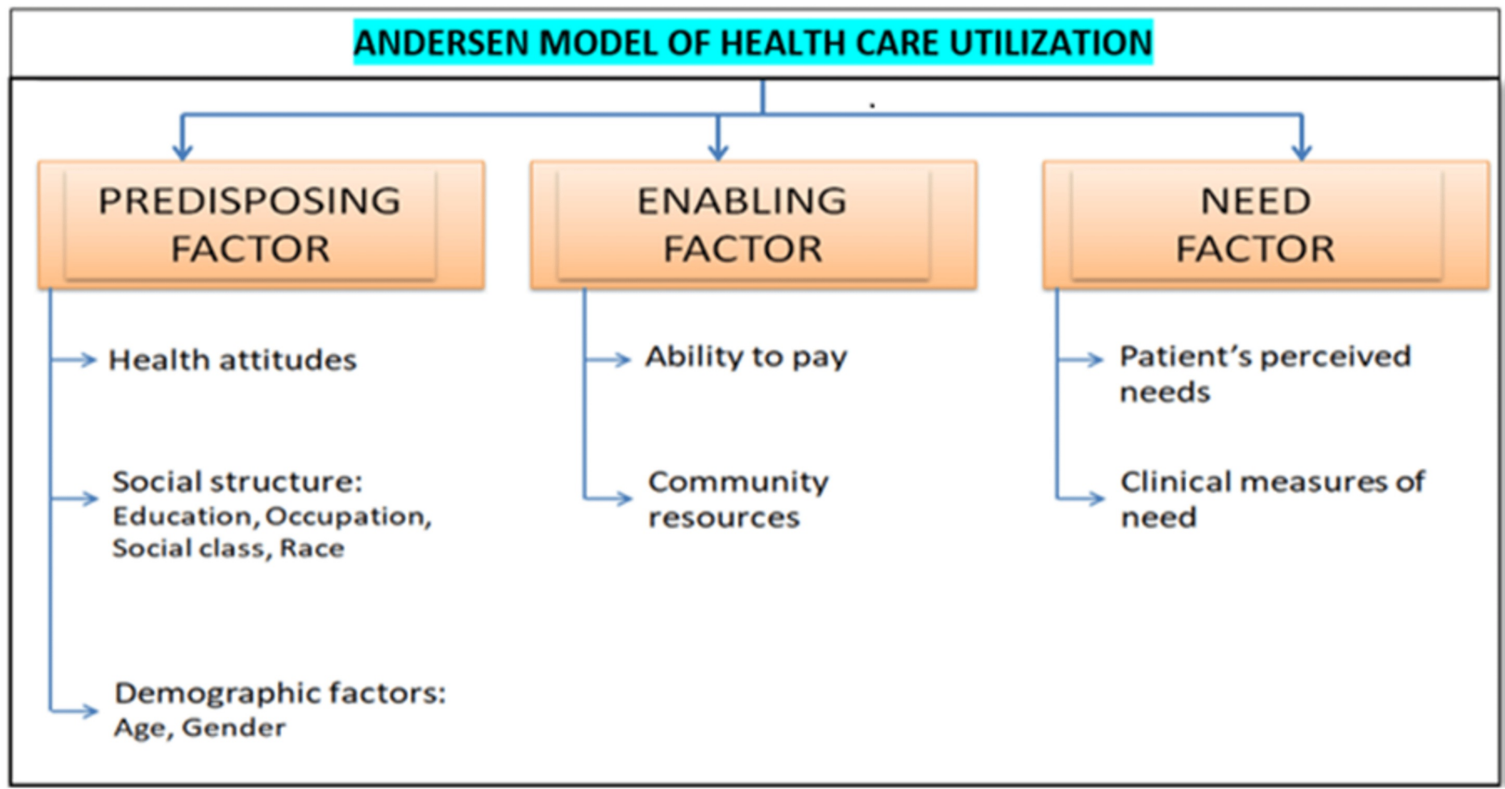
Andersen’s model of healthcare utilization

Developing an effective questionnaire begins with building a conceptual framework [5]. Various factors influence the health services used by an individual. This questionnaire attempted to determine the components that influence the utilization of dental services based on Andersen’s model of healthcare utilization (Figure 2). The determinants mainly include three factors: the predisposing factor, the enabling factor, and the need factor [6,7].

Predisposing Component

Certain people have a tendency to use services more frequently than others, and this tendency can be anticipated by personal traits that exist before certain illness episodes begin. Even if these traits do not directly cause the use of health services, those who possess some of these traits are more likely to do so [8]. The predisposing factors used for this study were education, occupation, age of first dental visit, last dental visit, healthcare preference, reasons for not visiting the dentist, presence of any deleterious habit, and demographic factors like age, gender, and marital status of the individual [9].

Enabling Component

People must have access to resources to use health services, even if they are inclined to do so. Enabling is described as a state that allows a family to act by a value or meet a need regarding the utilization of health services. The individual has access to resources for health services when certain conditions are met [10]. The enabling factors taken into consideration here were family resources such as income, number of family members, health insurance coverage level, and preferred method of payment and availability of dental care facilities [11].

Need Component

For the individual or his family to use health services, it is assumed that predisposing and enabling factors are present. The individual and his family must also perceive sickness or the likelihood of illness occurring. The most direct reason why people need health services is their level of illness [12]. This included the reasons that press an individual to utilize dental care services and the presence of other related medical conditions [13,14].

Conducting a literature review

This was done to find any existing questionnaires in that field that could be altered or tailored to serve the intended function. This aided in determining the construct of the questionnaire [4]. A thorough literature search was conducted before the study to locate the tools, the variables influencing dental care use, and the procedures associated with creating a questionnaire for medical research. The PubMed, EBSCO online library, and Google Scholar databases were searched using these keywords: questionnaire, dental utilization, Andersen model, item development, validity, and reliability. Relevant articles were thoroughly studied.

We did not identify any structured and validated questionnaire for dental healthcare utilization.

Conducting interviews among focus groups

After the literature study demonstrated the need for a new questionnaire and contributed to its definition, the next stage was to determine whether the construct’s conceptualization corresponds to how potential respondents perceive it [15]. A face-to-face interview and discussion were conducted with 40 volunteers. This mainly included patients visiting SRM Kattankulathur Dental College & Hospital, Chengalpattu, India, from May to June 2023. The goal of the interviews and focus group discussions was to urge the subjects to actively participate and interact. To begin with, sound listening to participant’s own words as they discussed the construct, with little to no prompting from the researcher, was done. The process continued until no new ideas were offered by the groups. Every conversation was noted and recorded. The results of the literature review and focus groups were then merged. This data was further used for item generation for the questionnaire.

Development of item

This stage entailed compiling a set of items in simple language that accurately expressed the questionnaire’s construct [4]. The original questionnaire was created in English, and it was modified to ensure consistency among the questions.

Pilot test of the questionnaire

A pilot test was conducted during the last phase of questionnaire creation. Finding such issues that respondents find unclear was the aim of the pilot testing. After completion, the final draft of the questionnaire on the variables impacting healthcare service utilization was ready for validity and reliability testing. The final data collection form comprised 24 sets of questions in three different parts. The first part contained 10 statements related to the Predisposing component of the Andersen model of healthcare utilization. The second component consisted of 12 statements related to the enabling component. The last part consisted of two statements regarding the need factors.

In this study, participants above the age of 18 were included. The recommended sample size was 120 subjects (a subject-to-item ratio of 1:5 was used) for pilot testing of the questionnaire. Data was collected from July to August 2023. This study received ethical approval from the Institutional Ethical Committee, SRM Medical College and Hospital (Ethical clearance number SRMIEC- ST0523-507) before the commencement of the study. Every questionnaire had an information sheet outlining its goals and objectives. Informed consent was obtained after the study’s purpose and methodology were presented.

Statistical analysis

Test-retest reliability and internal consistency reliability tests were used to assess the reliability of the questions [16,17]. Thirty randomly chosen individuals from the same sample were asked to complete the questionnaire four weeks later to conduct a test-retest reliability study. Five experts in the field of public health dentistry reviewed and evaluated the questionnaire’s face validity as well as its content validity before it was finalized. The IBM SPSS Statistics, version 22.0 (IBM Corp., Armonk, NY), was used for data collection and analysis. The frequency and percentage were calculated for each factor. The Cronbach’s alpha coefficient was used to assess the questionnaire’s reliability in terms of internal consistency and corrected item-total correlations. Test-retest reliability was also calculated using Cronbach’s alpha coefficient. The content validity ratio (CVR), which measures the essentiality of an item, was used to establish the validity [18]. Lawshe’s (1975) content validity codes were applied.

## Results

Among the 120 participants, there were 58 (48.3%) individuals from the 18-24 years age group, 41 (34.2 %) individuals from the 25-54 years age group, 14 (11.7 %) individuals from the 55-64 years age group, and seven (5.8 %) individuals above 65 years of age. Males were 66 (55%) and females were 53 (44.2%). Most participants belonged to the upper class, and most participants had an undergraduate degree (67.5%). Tables 1-3 show the predisposing, enabling, and need factors of the study participants. An item’s essentiality was gauged using CVR. The range of CVR is −1 to 1. Higher scores were a sign of more agreement among panelists. Thirty-four items had CVR generated for them. Of the 34 items, 10 were deemed not essential and eliminated. The CVR of 24 items was 1.00, while the remaining items received a score of 0.

**Table 1:** Predisposing characteristics of study participants

Question	Category	Coding	Frequency	Percent
Q1. Education: what is your educational level?	Post-graduate	1	17	14.2
	Under-graduate	2	81	67.5
	Completed schooling	3	19	15.8
	School dropout	4	2	1.7
	Illiterate	5	1	0.8
Q2. Occupation: what is your occupation?	Professional	1	43	35.8
	Semi-professional	2	13	10.8
	Clerical/shop/farmer	3	19	15.8
	Skilled worker	4	11	9.2
	Semi-skilled worker	5	6	5
	Unskilled worker	6	2	1.7
	Unemployed	7	26	21.7
Q3. Age of first dental visit: what was your age when you went to the dentist for the first time?	Children: 0-14 years	1	23	19.2
	Early working age: 18-24 years	2	66	35.8
	Prime working age: 25-54 years	3	22	18.3
	Mature working age: 55-64 years	4	7	5.8
	Elderly: 65 years and above	5	1	0.8
	Don’t remember	6	13	10.8
	Never been to the dentist before	7	11	9.2
Q4. Last dental visit: what was the last time when you visited your dentist?	Less than 6 months back	1	31	25.8
	6 months to 1 year back	2	13	10.8
	More than 1 year back	3	22	18.3
	Don't remember	4	30	25
	Never been to the dentist before	5	24	20
Q5. Healthcare preference: which modality of treatment do you prefer for oral health-related problems?	Ayurveda	1	16	13.3
	Homeopathy	2	7	5.8
	Allopathic	3	72	60
	Home remedy	4	15	12.5
	Others	5	10	8.3
Q6. Reasons for not visiting: what is the reason that prevents you from visiting your dentist?	Don’t have a dental problem	1	67	55.8
	No service available	2	1	0.8
	No dental service nearby	3	1	0.8
	No transportation available	4	1	0.8
	Can’t afford	5	8	6.7
	Afraid	6	17	14.2
	Too busy	7	17	14.2
	Any other reason	8	8	6.7
Q7. Habits: do you have any of these deleterious habits?	No deleterious habits	1	73	60.8
	Not willing to disclose	2	16	13.3
	Smoking	3	20	16.7
	Smokeless tobacco (Gutka, panparag, and paan masala)	4	6	5
	Alcohol	5	3	2.5
	Others (e-cigarette and vape)	6	2	1.7
Q8. Age: what is your age?	Early working age: 18-24 years	1	58	48.3
	Prime working age: 25-54 years	2	41	34.2
	Mature working age: 55-64 years	3	14	11.7
	Elderly: 65 years and above	4	7	5.8
Q9. Gender: what is your gender?	Male	1	66	55
	Female	2	53	44.2
	Transgender	3	1	0.8
	Not willing to disclose	4	Nil	Nil
Q10. Marital status: what is your marital status?	Married	1	42	35
	Unmarried	2	71	59.2
	Others (divorced, widowed, and separated)	3	7	5.8

**Table 2:** Enabling characteristics of study participants

Question	Category	Coding	Frequency	Percent
Q11. Income: what is your monthly family income in rupees?	≥185,895	1	34	28.3
	92,951-185,894	2	19	15.8
	69,535-92,950	3	33	27.5
	46,475-69,534	4	14	11.7
	27,883-46,474	5	5	4.2
	9308-27,882	6	13	10.8
	<9307	7	2	1.7
Q12. No. of family members: do you live in a joint or nuclear family?	Nuclear (2 generations/parents and children)	1	91	75.8
	Joint (>2 generations/grandparents, parents, and children)	2	29	24.2
Q13. Health insurance: do you have health insurance?	Yes	1	38	31.7
	No	2	65	54.2
	Planning to take	3	9	7.5
	Not willing	4	8	6.7
Q14. Preferred method of payment: how would you prefer to make payment for your treatment?	Single payment	1	76	63.3
	Installments	2	44	36.6
Q15. Locality: what kind of locality do you live in?	Rural	1	42	35
	Urban	2	78	65
Q16. Availability of dental care: what kind of dental health services are available near you?	Government hospital	1	22	18.3
	Private/corporate hospital	2	51	42.5
	Clinic	3	41	34.2
	None	4	3	2.5
	Don’t know	5	3	2.5
Q17. Time to reach: how much time do you need to reach your dental clinic?	Less than half an hour	1	59	49.2
	Half an hour to 1 hour	2	42	35
	More than 1 hour	3	8	6.7
	Can't say	4	11	9.2
Q18. Transport available: what type of transportation services are available to reach your dental clinic?	Public transport	1	51	42.5
	Private	2	58	48.3
	None	3	11	9.2
	Don’t know	4	Nil	Nil
Q19. Proximity of available dental services: how far is your dental clinic from your place?	Less than 1 km	1	35	29.2
	1-2 km	2	45	37.5
	More than 2 km	3	40	33.3
Q20. Waiting time at the dentist: what is the average time that you have to wait at the dental clinic before seeing your dentist?	Less than half an hour	1	26	21.7
	More than half an hour	2	42	35
	1 hour	3	29	24.2
	Can't say	4	23	19.2
Q21. Can you take leave from work/office to get your dental treatment done?	Yes	1	39	32.5
	No	2	30	25
	Sometimes	3	38	31.7
	Can’t say	4	13	10.8
Q22. Are you aware of the various dental treatment modalities available?	Yes	1	32	26.7
	No	2	54	45
	Have some idea	3	34	28.3

**Table 3:** Need characteristics of study participants

Question	Category	Coding	Frequency	Percent
Q23. Reason to visit: what is the reason for your dental visit?	General checkup	1	35	29.2
	Pain	2	38	31.7
	Other reasons: ceaning, alignment of teeth, replacement, oral malodor, etc.	3	47	39.2
Q24. Past medical history: are you suffering from any medical problems?	Yes	1	51	42.5
	No	2	69	57.5

Table 4 presents the findings of the internal consistency study for each item. Over four weeks, the test-retest reliability was examined in a pilot sample of 30 people. The correlation was between 78% and 85%. An acceptable Cronbach’s alpha value of 0.72 was discovered.

**Table 4:** Item-total statistics

	Scale mean if item deleted	Scale variance if item deleted	Corrected item-total correlation	Cronbach's alpha if item deleted
Q1	47.4	54.267	0.334	0.365
Q2	44.5	43.611	0.218	0.366
Q3	47.2	51.733	0.221	0.368
Q5	46.1	59.656	-0.096	0.44
Q6	45.5	47.611	0.052	0.462
Q7	47.8	53.956	0.471	0.356
Q8	46.9	54.989	0.373	0.369
Q9	47.7	63.567	-0.545	0.457
Q10	47.5	62.722	-0.532	0.447
Q11	45.9	43.878	0.343	0.308
Q12	48.1	56.767	0.378	0.384
Q13	47.7	55.344	0.213	0.382
Q14	47.9	57.878	0.153	0.399
Q15	47.6	59.6	-0.066	0.417
Q16	47.3	58.456	0.018	0.412
Q17	48	54.889	0.4	0.367
Q18	47.7	57.789	0.1	0.402
Q19	47.5	55.611	0.211	0.383
Q20	46.8	60.622	-0.145	0.444
Q21	47.2	57.289	0.097	0.401
Q22	47.5	54.056	0.402	0.36
Q23	47.3	55.789	0.237	0.382
Q24	47.5	62.278	-0.467	0.443

## Discussion

The utilization of healthcare services is synonymous with a good working healthcare system [19]. Andersen’s model, developed in the 1960s, is one of the most accepted models related to healthcare utilization, which includes predisposing, enabling, and need factors that influence the usage of health services. The utilization of dental health services varies considerably. To reduce disparities in oral health and overcome potential impediments, it can be helpful to identify the factors that influence the use of dental services.

In our study, it was discovered that the socio-demographic traits of the populace, such as age, education, and occupation, had an impact on the use of dental services. These results are consistent with research reports by Gambhir et al. [2], which stressed the need to educate individuals while also taking into account the unique factors limiting their behavior in order to encourage them. Better-educated participants are more likely to use dentist services. The use of dental care was higher among males and individuals aged 15-24. The results of this study contrast those of a study published by Pradeep et al. [20], which found that women used services at a higher rate than men.

There was a noteworthy distinction between the individuals in the lower-income group and those with greater family incomes. This result is consistent with that of Davidson and Andersen [7], which presents similar results. The study’s findings showed that place of residence and the ease of access to dental services were strongly correlated with the use of dental services, with those who lived in urban areas and close to dental facilities visiting the dentist more frequently than those in the other group.

The study’s findings indicate that there is a pressing need to inform the public about the value of dental health and how it relates to overall health [10]. The percentage of the population visiting the dentist for an immediately identifiable reason is more compared to those visiting for general dental check-ups. About 70.3% of respondents said that they only seek dental care when they feel it is necessary rather than as part of a routine general examination. This result is consistent with research conducted by Garcha et al. [21]. This may be because oral issues are not as serious as general health issues and are only sought after when symptoms develop.

The limitation of this questionnaire development study is that it included a larger number of young people. Most of the participants belonged to the higher-income category. The population cannot be represented by this small sample size. Furthermore, only those who were familiar with the English language were addressed; as a result, evaluation in the local language is advisable. Similarly, confirmatory factor analysis can be another suggested course of action.

## Conclusions

The questionnaire that was developed for the pilot study was a valid and reliable tool for identifying the factors that influence the use of dental care. Therefore, it can be a helpful tool for government entities and medical professionals conducting research to determine what obstacles prevent people from using dental treatments. All things considered, our results clearly imply that the 24-item scale, which is based on the Andersen model, is a legitimate and trustworthy tool for determining the variables affecting the use of dental healthcare. To assess the usability, generalizability, and application of this tool to various populations, further investigation is advised. The questionnaire’s extensive design emphasizes how safe it is to use in any population. This research yields positive results and provides evidence of a strong assembly of conceptually standardized components.
